# Association of Therapeutic Plasma Exchange-Treated Thrombotic Thrombocytopenic Purpura with Improved Mortality Outcome in End-Stage Renal Disease

**DOI:** 10.3390/diseases13080247

**Published:** 2025-08-05

**Authors:** Brenna S. Kincaid, Kiana Kim, Jennifer L. Waller, Stephanie L. Baer, Wendy B. Bollag, Roni J. Bollag

**Affiliations:** 1 Medical College of Georgia, Augusta University, Augusta, GA 30912, USAkiakim@augusta.edu (K.K.); stephanie.baer@va.gov (S.L.B.); 2 Department of Family and Community Medicine, Medical College of Georgia, Augusta University, Augusta, GA 30912, USA; jwaller@augusta.edu; 3 VA Augusta Health Care System, Augusta, GA 30904, USA; 4 Department of Physiology, Medical College of Georgia, Augusta University, Augusta, GA 30912, USA; 5 Department of Pathology, Medical College of Georgia, Augusta University, Augusta, GA 30912, USA

**Keywords:** hematology, nephrology, autoimmune, coagulopathy, plasmapheresis

## Abstract

**Background/Objectives**: Thrombotic thrombocytopenic purpura (TTP) is a microangiopathic hemolytic anemia exhibiting 90% mortality without prompt treatment. The aim of this study was to investigate the association of therapeutic plasma exchange (TPE)-treated TTP in end-stage renal disease (ESRD) patients with mortality, demographics, and clinical comorbidities. We queried the United States Renal Data System for ESRD patients starting dialysis between 1 January 2005 and 31 December 2018, using International Classification of Diseases (ICD)-9 and ICD-10 codes for thrombotic microangiopathy, with a TPE procedure code entered within 7 days. **Methods**: Cox proportional hazards models were used to assess mortality, adjusting for demographic and clinical factors. **Results**: Among 1,155,136 patients, increased age [adjusted odds ratio (OR) = 0.96, 95% confidence interval (CI): 0.94–0.96]; black race (OR = 0.67, CI: 0.51–0.89); and Hispanic ethnicity (OR = 0.43, CI: 0.28–0.66) were associated with a lower risk of TPE-treated TTP diagnosis, whereas female sex (OR = 1.59, CI: 1.25–2.02) and tobacco use (OR = 2.08, CI: 1.58–2.75) had a higher risk. A claim for TPE-treated TTP carried a lower risk of death (adjusted hazard ratio = 0.024, CI: 0.021–0.028). Female sex, black race, Hispanic ethnicity, and hypothyroidism were also associated with decreased all-cause mortality. **Conclusions**: These findings suggest that ESRD patients with TPE-treated TTP are significantly protected from mortality compared with ESRD patients without this diagnosis.

## 1. Introduction

Thrombotic thrombocytopenic purpura (TTP) is a rare hematologic condition associated with high morbidity and mortality. It occurs due to a decrease in the activity or absence of the enzyme *ADAMTS13*, a metalloprotease crucial to the cleavage of von Willebrand multimers. Congenital TTP is due to biallelic germline mutations in the *ADAMTS13* gene, while immune-mediated TTP develops due to an inhibitor or autoantibody-mediated destruction of the *ADAMTS13* protein [1]. In both cases, the condition leads to widespread thrombotic coagulopathy and bleeding diathesis. The most common risk factors for the development of immune-mediated TTP are antiplatelet drugs, immuno-suppressive agents, human immunodeficiency virus (HIV), combination oral contraceptives, and pregnancy [2]. TTP is rare, with an overall incidence of 1.5–6 cases per million adults annually [3]. Demographically, female sex (with an incidence 2–3 times that of men) and African American race (with an eight-fold higher incidence than Caucasian race) are well known associations for TTP incidence in the general population [3,4]; pregnancy can also sometimes precipitate TTP [3].

Most patients with TTP present with a microangiopathic hemolytic anemia with thrombocytopenia, which can rapidly develop into a thrombotic or bleeding diathesis; therefore, a high index of suspicion must be maintained depending on the clinical picture [5]. Additionally, laboratory diagnosis depends on establishing diminished *ADAMTS13* activity; therefore, diagnostic certainty usually cannot be ensured in a timely manner. Since this is a hematologic emergency, diagnosis is often made based on clinical judgment [6]. Diagnosis can be assisted with the PLASMIC score, which predicts the likelihood of *ADAMTS13* activity as being less than or equal to 10% of normal activity [7]. This index assists the clinician in forming a presumptive diagnosis of TTP to initiate rapid treatment. A score greater than 5 suggests a high probability of TTP. This score is validated with a sensitivity of 99% and a specificity of 57% [8]. However, other studies have shown that the PLASMIC score has lower sensitivity and specificity than alternative diagnostic methods such as absolute immature platelet count (A-IPC) [9].

Prompt diagnosis and treatment is crucial, as time to death in disease without proper treatment has been reported to be as short as 4 days [10]. The mainstay of treatment is plasma infusion or plasma exchange for both types of TTP, with corticosteroid therapy for immune-mediated TTP. Steroids assist in treatment by decreasing autoantibody production, while plasma exchange removes autoantibodies and large von Willebrand factor multimers from circulation and replaces deficient ADAMTS13 protein activity [11]. Treatment is given daily until remission. The remission of TTP is defined as a sustained clinical response (normalized platelet count) with either no therapeutic plasma exchange (TPE) and no anti-VWF therapy after 30 days, or the normalization of ADAMTS13 levels [12]. To prevent relapse, there are also second-line treatments including rituximab, splenectomy, cyclosporine, cyclophosphamide, and vincristine. The addition of rituximab has been shown to decrease the duration of plasma exchange needed to achieve remission [13]. The use of recombinant *ADAMTS13* infusion has not been approved as of 2023. A new agent, caplacizumab, has recently become available, which attaches to von Willebrand factor and prevents platelet adhesion. Caplacizumab is effective in decreasing the hemolysis associated with TTP but does not affect autoantibody production [14].

Relapse is still common, even with treatment, at 40% in immune TTP and 69% in congenital TTP. Furthermore, since both chronic kidney disease/ESRD and TTP can lead to anemia [15], it is possible that these two conditions together could result in worse outcomes—that is, greater mortality. Therefore, it is of interest to evaluate mortality in patients with TTP who were treated with TPE and who are on long-term hemodialysis. 

## 2. Materials and Methods

### 2.1. Study Design

The purpose of this study was to determine whether TTP is an independent risk factor for mortality in a retrospective cohort study by interrogating the USRDS administrative database [16] using International Classification of Disease (ICD)-9 and -10 codes, comparing the mortality of those ESRD patients with a diagnosis of TPE-treated TTP and those without, using the computer program SAS 9.4. The Augusta University Institutional Review Board (AU IRB) deemed that this research did not constitute human subjects research (reference #1592144-1, dated 25 March 2022).

### 2.2. Study Cohort

The study sample comprised all ESRD patients in the USRDS database who were between the ages of 18 and 100 years at the time of the start of dialysis and who began therapy between 1 January 2005 and 31 December 2018; these inclusion criteria allowed for follow-up and accrual of claims to determine the outcome (mortality) and independent variables that were controlled. Subjects with missing information, such as sex, race, ethnicity, age, or no or unknown incident dialysis modality or access type, were excluded from the study cohort. The number of included ESRD subjects totaled 1,155,136. These patients exhibited an average age of 63.8 (SD = 15.0); 44% were female; 27% were of black race; 7% were of other race; and 16% were Hispanic. Nearly all were on hemodialysis and the majority used a catheter for their access type (81%). Among the included cohort, 12% had hypothyroidism, 1.3% had illicit drug use, 13% used tobacco and 4% were alcohol-dependent.

### 2.3. Primary Independent Variable

To determine whether TTP was an independent risk factor for mortality in the ESRD population, we first queried our variable of interest, a TTP diagnosis. As the ICD-9 and -10 codes for TTP encompass other thrombotic microangiopathies, for this study we defined a patient with TTP as having at least one diagnosis of thrombotic micro-angiopathy with a TPE treatment procedure code within 7 days of that diagnosis (TPE-treated TTP). We note that this combination approach has been shown to improve the positive predictive value of claims data, as validated by medical record data abstraction [17]. Claims were identified in either the hospital, physician/supplier, or detailed claims data files using the ICD-9-CM and ICD-10-CM diagnosis and Current Procedural Terminology (CPT) and Healthcare Common Procedure Coding System (HCPCS) procedure codes (Supplementary Table S1). Noted as a limitation in Section 4.4, this strategy of identifying TTP could introduce bias related to the clinical decision to treat TTP with TPE; thus, suspected TTP patients who were too ill or lacked vascular access to undergo TPE might have been included in the control group and may have had greater mortality.

### 2.4. Outcome Variable

The primary outcome of interest in this study was time to death in years. Time to death was defined for all subjects as the number of years from the start of dialysis until death, or to 31 December 2019, for those who did not die. Patients alive at the end of the study period were considered censored observations.

### 2.5. Other Independent Variables

Demographic variables were obtained from the Centers for Medicare & Medicaid Services (CMS) Medical Evidence Form 2728 and included sex, race, ethnicity, age at dialysis initiation, and incident dialysis modality and access type. Comorbid conditions that were included as covariates included hypothyroidism, illicit drug use, alcohol dependence, tobacco use, and the Charlson comorbidity index (CCI), an overall measure of the number and severity of comorbidities [18]. ICD-9 and ICD-10 codes recorded in claims data were used to determine the presence of comorbid diagnoses (Supplementary Table S2).

### 2.6. Statistical Analysis

All statistical analyses were performed using SAS 9.4; statistical significance was assessed using an alpha level of 0.05. Descriptive statistics were determined overall by TPE-treated TTP, and by mortality status.

Logistic regression was used to examine the association of the covariates and to determine potential confounding of the demographic and clinical parameters with TPE-treated TTP. Initially, each of the independent variables was examined in a simple logistic regression model. Then, a backward model-building strategy was used to create a multivariable logistic regression model. With this strategy, independent variables with non-significant *p*-values, starting with the largest, were serially removed, and the model fit was determined. If the altered model provided a better fit to the data based on a −2log(likelihood) test and Akaike’s Information Criterion, the variable remained excluded from the model. Otherwise, the variable was restored and the next independent variable was removed and studied for exclusion. The final model contained all statistically significant independent variables or those that improved model fit. Adjusted odds ratios (OR) and 95% confidence intervals (CI) were determined.

Propensity scores for a diagnosis of TPE-treated TTP were determined using all the demographic and clinical parameters discussed above. Inverse propensity scores were adjusted back to the sample size in each TPE-treated TTP group and were subsequently used as a weight in the Cox proportional hazards model building.

A possible relationship between TPE-treated TTP and survival was analyzed descriptively using Kaplan–Meier survival curves comparing patients with and without TPE-treated TTP using a log-rank test. To control for potential covariates, Cox proportional hazards (CPH) modeling, with the adjusted inverse propensity score weights determined as above, was used to examine the association between TPE-treated TTP and mortality, controlling for the delineated demographic and comorbid parameters. TPE-treated TTP and all potential covariates were examined in simple CPH models. Then, a similar backward model-building process was conducted, as above, to arrive at a final multivariable model. Hazard ratios (HR) and 95% confidence intervals (CI) were ascertained and reported.

## 3. Results

### 3.1. Factors Associated with a TPE-Treated TTP Diagnosis

Of the 1,155,136 subjects with ESRD included in the analysis, 66% died during the study period; the average time to death was 3.5 months. Supplementary Table S3 provides the descriptive statistics by TPE-treated TTP status as well as the results of the simple logistic regression analysis. Supplementary Table S4 gives the results for the full and final multiple logistic regression model on TPE-treated TTP; Supplementary Figure S1 shows the corresponding forest plot of the final multiple logistic regression model, indicating factors associated with this diagnosis.

Increasing age (OR = 0.96, 95% CI: 0.94–0.96), black race compared to white race (OR = 0.67, 95%CI: 0.51–0.89), and Hispanic ethnicity (OR = 0.43, 95% CI: 0.28–0.66) were associated with a lower risk of TPE-treated TTP. Female sex (OR = 1.59, 95% CI: 1.25–2.02), tobacco use (OR = 2.08, 95% CI: 1.58–2.75), and increasing CCI (OR = 1.13, 95% CI: 1.10–1.17) were associated with an increased risk of TPE-treated TTP. Due to USRDS reporting regulations that protect patient confidentiality, we cannot provide exact numbers or percentages; however, it should be noted that a large percentage of ESRD patients identified with TPE-treated TTP were also diagnosed with hypothyroidism. The low frequency of treated TTP patients without hypothyroidism also precluded a determination of the odds ratio.

### 3.2. Association Between TPE-Treated TTP and Mortality

**Figure 1:**
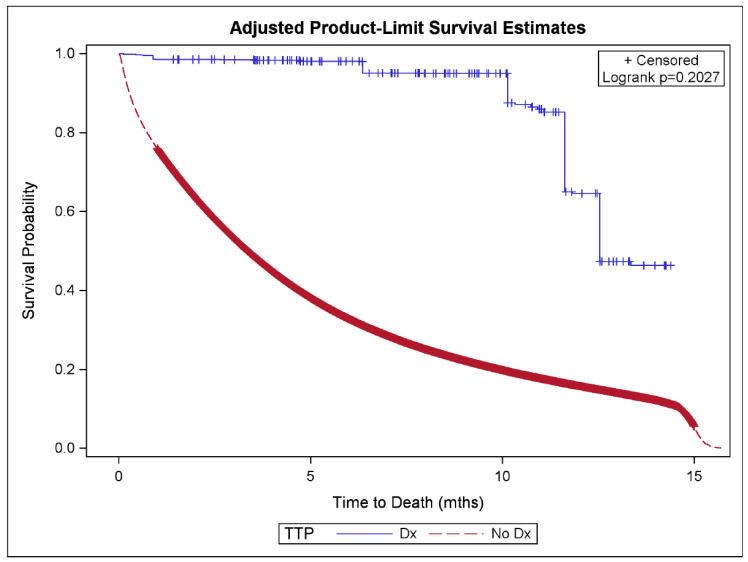
Kaplan–Meier survival curve on mortality by TTP plus treatment status using adjusted inverse propensity score weights. Abbreviations used: TTP = thrombotic thrombocytopenic purpura, Dx = diagnosis.

**Figure 2:**
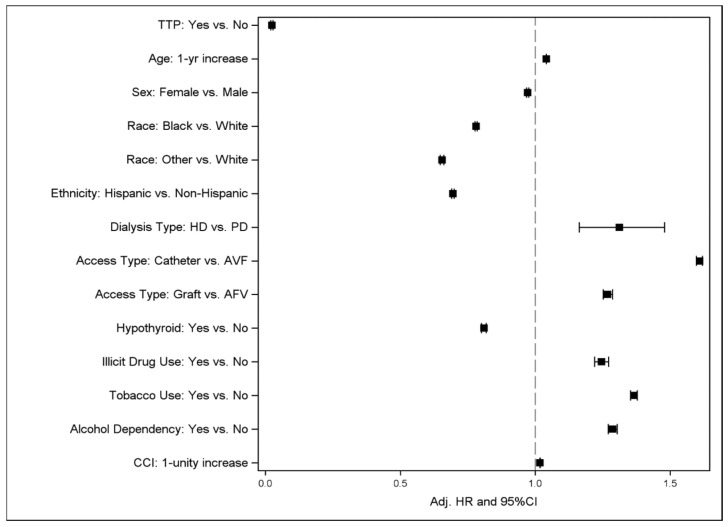
Forest plot of final multiple CPH model of TTP plus treatment on mortality controlling for various covariates and confounders. Abbreviations used: TTP = thrombotic thrombocytopenic purpura, HD = hemodialysis, PD = peritoneal dialysis, AVF = arteriovenous fistula, CCI = Charlson Comorbidity Index.

Table 1 shows the descriptive statistics by mortality status and the hazard ratios (HR) and 95% CI from the simple and final CPH models. Figure 1 illustrates the Kaplan–Meier curve for mortality by TPE-treated TTP; Figure 2 shows a forest plot of the final multivariable CPH model of TPE-treated TTP on mortality. 

TPE-treated TTP was associated with significantly decreased all-cause mortality in both the simple model (HR = 0.033, 95% CI: 0.028–0.038) and the final multivariable model (HR = 0.024, 95% CI: 0.021–0.028). All other demographic and clinical covariates were associated with an altered risk of mortality. Increasing age, hemodialysis, catheter access or graft access compared to arteriovenous fistula (AVF) access, illicit drug use, tobacco use, and alcohol dependency were significantly associated with an increased risk of mortality. Female sex, black race or other race compared to white race, Hispanic ethnicity, and hypothyroidism were associated with decreased all-cause mortality.

## 4. Discussion

### 4.1. Demographics

The goal of our study was to describe mortality in patients with ESRD and comorbid TTP who were treated with TPE compared to that of patients with ESRD without a diagnosis of TPE-treated TTP. There were 1,155,136 subjects in the USRDS database who started dialysis between 1 January 2005 and 31 December 2018 and were included in our final analysis. Of these subjects, 0.02% had a comorbid diagnosis of TPE-treated TTP. This prevalence is comparable to current studies in the literature that have determined a suspected annual incidence of 1.5 to 6 cases per million people [3].

The average age of a patient with ESRD in our sample was 63.8, while the average age of a patient with ESRD and TPE-treated TTP was 54.4 (*p* <0.001). TTP is known to be more common in younger patients, with a median age of onset in the fourth decade of life [19]. In our patient population, patients with TTP were older than expected based on a literature review, which may be attributable to the older demographic of patients with ESRD. TTP is also known to have a female predominance [20], which was true in our subpopulation as well, with females comprising 53.9% of ESRD patients with TPE-treated TTP and 44.3% of ESRD patients without the diagnosis (*p* = 0.0016). A previous study has established a higher risk of TTP in the black population [4]. However, our cohort showed no significant difference in the percentage of the TPE-treated TTP patients of black race versus percentage of those of black race in the ESRD population without such a diagnosis.

Both immune-mediated and congenital TTP are thought to develop due to a triggering event. Pregnancy is a well-described common trigger for TTP development. Infection and inflammatory states are also thought to be associated with TTP [21]. In our population, patients with TPE-treated TTP were more likely to have illicit drug use (3.7% vs. 1.3% in the ESRD population without the TPE-treated TTP diagnosis, *p* = 0.0012). They were also more likely to have a diagnosis of tobacco use (26.8% in TPE-treated TTP vs. 12.5% in ESRD only, *p* < 0.0001). Current studies in the literature do not describe a strong correlation between tobacco or illicit drug use and TTP development. However, inflammatory stress may promote microvascular thrombosis through endothelial injury, which could trigger TTP. Therefore, the increased tobacco use among patients with TPE-treated TTP, compared to those without TTP, could contribute to the development of such a disease state.

The CCI is a scoring system used to estimate ten-year survival based on age and a variety of comorbidities, including congestive heart failure, peripheral vascular disease, diabetes mellitus, and chronic kidney disease. It is important to note that additional weight (2 points) is given to the presence of chronic kidney disease requiring dialysis. Therefore, the minimum CCI score for any patient in the USRDS database is 2. In our study, the mean Charlson Comorbidity Index for patients with ESRD without TPE-treated TTP was 5.8, whereas the mean CCI for patients with treated TTP and ESRD was 7.3 (*p* < 0.0001). A CCI of 6 is correlated with 2% ten-year survival, while a CCI of 7 is correlated with 0% estimated ten-year survival. This would imply that patients with TTP and ESRD could be expected to have significantly lower survival rates based on the comparison of CCI scores, if those scores were not controlled for.

### 4.2. Comorbidities

In the literature, comorbidities associated with TTP are primarily anecdotal since, to our knowledge, no extensive investigation of this patient population has been performed to date. However, our study found an interesting correlation between hypothyroidism and TPE-treated TTP that warrants further analysis. Among all patients with ESRD, 12% had hypothyroidism. Among women with ESRD and TTP, a large percentage had comorbid hypothyroidism; in accordance with USRDS rules, exact numbers or percentages could not be included to protect patient anonymity. We postulate that this result may point to a previously undescribed association between TPE-treated TTP and hypothyroidism. However, this association may be partly explained by the predominance of autoimmune conditions among young women, including TTP and Hashimoto’s thyroiditis [20,22]. Of relevance to this association is that although the well-curated registry of patients with hereditary TTP indicates autoimmune disease association, including hypothyroidism, it is rare in the congenital population [23]. While the USRDS does not collect genetic data, precluding an assessment of hereditary TTP cases in the current cohort, hereditary TTP is exceedingly rare. Thus, the association of TTP with hypothyroidism in the current analysis most likely reflects a common autoimmune pathway predisposing to hypothyroidism and immune-mediated TTP. Our result is also of interest as there is a well-described predominance of TTP among young pregnant women. It is thought that patients with a genetic predisposition for TTP may be triggered by the hormonal changes in pregnancy [24]. This association raises an interesting premise, suggesting a possible endocrine component of TTP development. It has been well-established that there is a worsening of hypothyroidism during pregnancy related to estrogen-dependent increases in thyroglobulin binding hormone [25]. Therefore, it is possible that there is an association between hormones of the hypothalamic–pituitary axis, particularly with changes during pregnancy, and the development of TTP. This result further implies that there may be a way to risk-stratify young women with hypothyroidism for increased surveillance for the possible development of TTP.

### 4.3. Mortality

Interestingly, we found that the diagnosis of TPE-treated TTP in the ESRD population was associated with decreased all-cause mortality in both the simple and final multivariable models. Demographic variables, such as female sex, black race and other race compared to white race, were also associated with decreased all-cause mortality, as was hypothyroidism. Because of TTP’s high mortality, we expected a diagnosis of TTP to be associated with higher mortality in ESRD patients in the USRDS database. One possible explanation for the lowered risk of death for patients with TTP treated with TPE is that TTP has a definitive treatment. Treatment with TPE significantly improves mortality. Without treatment, mortality in TTP is 90%; with treatment, mortality improves to 10 to 15% [8]. With newer tailored therapies for TTP in conjunction with plasma exchange, this mortality is likely to decrease further. Therefore, patients diagnosed with TTP receiving appropriate treatment with plasma exchange show a reduction in the elevated risk of mortality associated with TTP.

As mentioned above, our results showed a high co-occurrence of hypothyroidism and TPE-treated TTP. A possible explanation for the decreased risk of death in those with TPE-treated TTP is that, as mentioned above, the average age of an ESRD patient with TPE-treated TTP in our sample was 54.4 ±18.2, while the average age of patients with ESRD was 63.8 ± 15.9. The population of ESRD patients with treated TTP, being significantly younger than the overall ESRD patient sample, may have contributed to the improved survival in those with treated TTP. The multivariable model should account for age; however, this is not easily accommodated in a Kaplan–Meier analysis.

### 4.4. Limitations

Our study utilized administrative data from the USRDS. The USRDS is a national data system that collects, analyzes, and distributes information about chronic kidney disease and ESRD. However, there are many limitations in the dataset. First, for the purpose of the current analysis, there were no ICD-9 or -10 codes specifically for TTP, although one has been added for the 2025 version of ICD-10-CM codebook (D69.3 for immune-mediated TTP). Indeed, many patients with TTP are initially diagnosed with microangiopathic hemolytic anemia and are only diagnosed with TTP after delayed laboratory results of deficient *ADAMTS13* activity are received. Therefore, to improve the accuracy of the TTP diagnosis, we defined our subpopulation as patients with an ICD-9 or ICD-10 code for thrombotic microangiopathy with a therapeutic plasmapheresis/plasma exchange treatment procedure code within 7 days of that diagnosis. Patients who never received therapeutic plasma exchange were likely excluded in this analysis because they never received the correct diagnosis and/or did not receive treatment within 7 days, perhaps because they were medically unstable or lacked vascular access for plasma exchange. These cases may then have contributed to the higher mortality rate in the general ESRD population, possibly introducing bias into the analysis. However, since it is a claims database, the USRDS dataset provides no insight into clinical decision making and we are unable to address this potential source of bias. In addition, we were unable to account for any errors in diagnosis or coding. Since the USRDS database also does not provide laboratory results, we were also not able to specifically select patients with decreased *ADAMTS13* enzyme activity or low platelet counts. In addition, the patients included in the present study predate the general availability of novel pharmacological treatments such as caplacizumab. Finally, this study was retrospective, which complicates establishing a cause-and-effect relationship between TPE-treated TTP in ESRD patients and mortality.

## 5. Conclusions

Our findings suggest that ESRD patients with TTP who are treated with therapeutic plasmapheresis/plasma exchange are protected from mortality compared with the comparison cohort of ESRD patients lacking the diagnosis. This protection may be attributable to the existence of a well-established treatment regimen for this condition, as compared to other disease processes with more complex and nuanced treatments. Additionally, we found a high incidence of comorbid hypothyroidism in patients diagnosed with TPE-treated TTP. Thus, future research to assess potential endocrine associations with TTP may be warranted, as are studies to determine the effects of new drugs, like caplacizumab, on all-cause mortality associated with TTP.

## Figures and Tables

**Table 1:** Descriptive statistics by mortality, and simple and final CPH models on mortality, weighted using adjusted inverse propensity score weights *.

Variable	Level	Descriptive Statistics		Simple CPH Models			Final CPH Model		
		Died	Alive	HR	95% CI	*p*-Value	HR	95% CI	*p*-Value
Time to Death or Follow-Up—mean (SD)		2.6 (2.6)	5.3 (3.6)						
Main Independent Variable									
TTP plus treatment—(%)	Yes	0.02	0.8	0.033	0.028–0.038	<0.0001	0.024	0.021–0.028	<0.0001
	No	99.98	99.2	1.00			1.00		
Demographic or Clinical Covariates									
Age—mean (SD)		67.7 (13.4)	56 (14.9)	1.042	1.042–1.043	<0.0001	1.040	1.040–1.040	<0.0001
Sex (%)	Female	45.6	41.4	1.06	1.06–1.07	<0.0001	0.97	0.97–0.98	<0.0001
	Male	54.4	58.6	1.00			1.00		
Race (%)	Black	24.9	31.8	0.73	0.72–0.73	<0.0002	0.78	0.78–0.78	<0.0001
	Other	5.5	8.4	0.66	0.65–0.66		0.65	0.65–0.66	
	White	69.6	59.8	1.00			1.00		
Ethnicity (%)	Hispanic	13.4	20.9	0.67	0.66–0.67	<0.0001	0.69	0.69–0.70	<0.0001
	Non-Hispanic	86.6	79.1	1.00			1.00		
Dialysis Type (%)	HD	99.97	99.9	1.58	1.40–1.78	<0.0001	1.31	1.16–1.48	<0.0001
	PD	0.03	0.1	1.00			1.00		
Access Type (%)	Catheter	83.5	76.8	1.48	1.47–1.49	<0.0001	1.61	1.60–1.62	<0.0001
	Graft	3.3	3.1	1.33	1.31–1.34		1.27	1.27–1.25	
	AVF	13.2	20.1	1.00			1.00		
Hypothyroidism (%)	Yes	14.8	6.8	1.19	1.19–1.20	<0.0001	0.81	0.80–0.82	<0.0001
	No	85.3	93.2	1.00			1.00		
Illicit Drug Use (%)	Yes	1.3	1.4	0.77	0.75–0.78	<0.0001	1.25	1.22–1.27	<0.0001
	No	98.7	98.6	1.00			1.00		
Tobacco Use (%)	Yes	14.9	8.5	1.20	1.19–1.21	<0.0001	1.37	1.35–1.38	<0.0001
	No	85.1	91.5	1.00			1.00		
Alcohol Dependent (%)	Yes	4.1	3.2	1.08	1.07–1.10	<0.0001	1.29	1.27–1.30	<0.0001
	No	95.9	96.8	1.00			1.00		
CCI—mean (SD)		6.7 (4)	3.8 (3.6)	1.055	1.054–1.056	<0.0001	1.017	1.017–1.018	<0.0001

* Abbreviations used: CPH = Cox proportional hazards, TTP = thrombotic thrombocytopenic purpura, HR = hazard ratio, CI = confidence interval, SD = standard deviation, HD = hemodialysis, PD = peritoneal dialysis, AVF = arteriovenous fistula, CCI = Charlson Comorbidity Index.

## Data Availability

The data underlying this article were provided by the USRDS under a data use agreement; data may be obtained from the USRDS upon establishment of a data use agreement. The interpretation and reporting of these data are the responsibility of the author(s) and in no way should be seen as an official policy or interpretation of the U.S. government. The contents of this article do not represent the views of the Department of Veterans Affairs or the United States Government.

## Supplementary Materials

The following supporting information can be downloaded at: https://www.mdpi.com/article/10.3390/diseases13080247/s1, Table S1: ICD-9, ICD-10, CPT and HCPCS codes used for the diagnosis of thrombotic thrombocytopenic purpura (TTP) and therapeutic plasma exchange (TPE); Table S2: ICD-9 and ICD-10 diagnosis codes for controlled comorbidities included in the Charleson Comorbidity Index and other covariates, Table S3: Descriptive statistics by TTP status, and simple logistic regression results on TTP plus treatment; Table S4: Full and final logistic regression models on TTP plus treatment. Figure S1: Forest plot of final multiple logistic regression model on TTP plus treatment. Abbreviation used: CCI=Charlson Comorbidity Index.

## Abbreviations

The following abbreviations are used in this manuscript:

AVF	Arteriovenous Fistula
CCI	Charleson Comorbidity Index
CI	95% Confidence Interval
CMS	Centers for Medicare & Medicaid Services
CPH	Cox Proportional Hazards
CPT	Current Procedural Terminology
ESRD	End-stage Renal Disease
HCPCS	Healthcare Common Procedure Coding System
HIV	Human Immunodeficiency Virus
HR	Hazard Ratio
ICD	International Classification of Disease
OR	Adjusted Odds Ratio
TPE	Therapeutic Plasma Exchange
TTP	Thrombotic Thrombocytopenic Purpura
USRDS	United States Renal Data System
