# Supplementary material for: Association of Therapeutic Plasma Exchange-Treated Thrombotic Thrombocytopenic Purpura with Improved Mortality Outcome in End-Stage Renal Disease
Source: Diseases. 2025 Aug 5;13(8):247. doi: 10.3390/diseases13080247 (PMC12385575; doi:10.3390/diseases13080247)
Supplement: Supplementary file 1 [file diseases-13-00247-s001.zip › diseases-3780688-supplementary.pdf]

Supplementary Materials for

Association of Therapeutic Plasma Exchange-treated Thrombotic Thrombocytopenic Purpura with Improved Mortality Outcome in End-Stage Renal Disease

by

Brenna S. Kincaid[1], Kiana Kim[1], Jennifer L. Waller[2], Stephanie L. Baer[1,3], Wendy B. Bollag[3,4,*], and Roni J. Bollag[5,*]

Supplementary Table S1. ICD-9, ICD-10, CPT and HCPCS codes used for the diagnosis of thrombotic thrombocytopenic purpura (TTP) and therapeutic plasma exchange*

Diagnosis	ICD-9	ICD-10	HCPCS	CPT
TTP (Thrombotic Microangiopathy)	446.6	M31.1, M31.19		
Therapeutic Plasmapheresis/Plasma Exchange			6A550Z3, 6A551Z3	99.71, 36514

* Abbreviations used: ICD = International Classification of Disease, CPT = Current Procedural Terminology, HCPCS = Healthcare Common Procedure Coding System, TTP = thrombotic thrombocytopenic purpura

Supplementary Table S2. ICD-9 and ICD-10 diagnosis codes for controlled comorbidities included in the Charleston Comorbidity Index and other covariates*

Variable	ICD-9 Codes	ICD-10 Codes
Acute MI	410, 412	I21, I22, I252
Congestive Heart Failure	428	I50
Peripheral Vascular Disease	441, 4439, 7854, V434	I71, I790, I739, R02, Z958, Z959
Cerebral Vascular Disease	430–438	I60, I61, I62, I63, I65, I66, G450, G451, G452, G458, G459, G46, I64, G454, I670, I671, I672, I674, I675, I676, I677 I678, I679, I681, I682, I688, I69
Dementia	290	F00, F01, F02, F051
Pulmonary Disease	490, 491, 492, 493, 494, 495, 496, 500, 501, 502, 503, 504, 505	J40, J41, J42, J44, J43, J45, J46, J47, J67, J44, J60, J61, J62, J63, J66, J64, J65
Connective Tissue Disorder or Rheumatologic Disease	7100, 7101, 7104, 7140, 7141, 7142, 71481(now 5171), 725	M32, M34, M332, M053, M058, M059, M060, M063, M069, M050, M052, M051, M353
Peptic Ulcer	531, 532, 533, 534	K25, K26, K27, K28
Liver Disease	5712, 5714, 5715, 5716	K702, K703, K73, K717, K740, K742, K746, K743, K744, K745

Variable	ICD-9 Codes	ICD-10 Codes
Diabetes	2500, 2501, 2502, 2503, 2507	E109, E119, E139, E149, E101, E111, E131, E141, E105, E115, E135, E145
Diabetes with Complications	2504, 2505, 2506	E102, E112, E132, E142 E103, E113, E133, E143 E104, E114, E134, E144
Paraplegia	342, 3441	G81 G041, G820, G821, G822
Renal Disease	582, 5830, 5831, 5832, 5833, 5835, 5836, 5837, 5834, 585, 586, 588	N03, N052, N053, N054, N055, N056, N072, N073, N074, N01, N18, N19, N25
Cancer	140-172, 174-195, 200, 201, 202, 203, 204, 205, 206, 207, 208	C0, C1, C2, C3, C40, C41, C43, C45, C46, C47, C48, C49, C5, C6, C70, C71, C72, C73, C74, C75, C76, C80, C81, C82, C83, C84, C85, C883, C887, C889, C900, C901, C91, C92, C93, C940, C941, C942, C943, C9451, C947, C95, C96
Metastatic Cancer	196, 197, 198, 1990, 1991	C77, C78, C79, C80
Severe Liver Disease	5722, 5723, 5724, 5728	K729, K766, K767, K721
HIV/AIDS	042, 043, 044	B20, B21, B22, B23, B24
**Other Covariates**		
Hypothyroidism (Hashimoto's Thyroiditis)	245.2	E06.3
Multiparity	V61.5	Z64.1
Illicit Drug Use	304.0, 304.2, 304.3, 305.2, 305.5, 305.6	F12.20, F12.10, F12.11, F14.10, F14.11, F14.20, F14.21

* Abbreviations used: ICD = International Classification of Disease, MI = Myocardial infarction, HIV = Human immunodeficiency virus, AIDS = Acquired immunodeficiency syndrome

Supplementary Table S3. Descriptive statistics by TTP status, and simple logistic regression results on TTP plus treatment[†]

Variable	Level	Overall	TTP				
			Yes N=269 (0.02%)	No N=1,154,867 (99.98%)	OR	95% CI	p-value
Age – mean (SD)		63.8 (15.0)	54.4 (18.2)	63.8 (15.9)	0.96	0.957-0.971	<0.0001
Sex – n (%)	Female	511640 (44.3)	145 (53.9)	511495 (44.3)	1.47	1.16-1.87	0.0016
	Male	643496 (55.7)	124 (46.1)	643372 (55.7)	1.00		
Race – n (%)	Black	315671 (27.3)	78 (29.0)	315593 (27.3)	1.10	0.84-1.44	0.7342
	Other	74903 (6.5)	19 (7.1)	74884 (6.5)	1.13	0.70-1.81	
	White	764562 (66.2)	172 (63.9)	764390 (66.2)	1.00		
Ethnicity – n (%)	Hispanic	184675 (16.0)	24 (8.9)	184651 (16.9)	0.52	0.34-0.78	0.0019
	Non-Hispanic	970461 (84.0)	245 (91.1)	970216 (84.9)	1.00		
Dialysis Type – n (%)	HD	1154567 (99.9)	NR	NR			
	PD	569 (0.1)	NR	NR			
Access Type – n (%)	Catheter	937453 (81.2)	NR	NR	1.24	0.87-1.77	0.4765
	Graft	37436 (3.2)	NR	NR	1.10	0.51-2.37	
	AVF	180247 (15.6)	NR	NR	1.00		
Hypo-thyroidism – n (%)	Yes	139745 (12.1)	NR	NR			
	No	1015391 (87.9)	NR	NR			
Illicit Drug Use – n (%)	Yes	15479 (1.3)	NR	NR	2.85	1.51-5.35	0.0012
	No	1139657 (98.7)	NR	NR	1.00		
Tobacco Use – n (%)	Yes	144641 (12.5)	72 (26.8)	144569 (12.5)	2.55	1.95-3.35	<0.0001

Variable	Level	Overall	TTP Yes N=269 (0.02%)	TTP No N=1,154,867 (99.98%)	OR	95% CI	p-value
	No	1010495 (87.5)	197 (73.2)	1010298 (87.5)	1.00		
Alcohol Dependent – n (%)	Yes	43675 (3.8)	13 (4.8)	43662 (3.8)	1.29	0.74-2.26	0.3659
	No	1111461 (96.2)	256 (95.2)	1111205 (96.2)	1.00		
CCI – mean (SD)		5.8 (4.1)	7.3 (3.0)	5.8 (4.1)	1.10	1.07-1.13	<0.0001
Mortality – n (%)	Died	767116 (66.4)	153 (56.9)	766963 (66.4)			
	Alive	388020 (33.6)	116 (43.1)	387904 (33.6)			
Cause of Death – n (%)	Cardiac	282470 (24.5)					
	Endocrine	61 (0.0)					
	Gastro-Intestinal	4933 (0.4)					
	Infection	64249 (5.6)					
	Liver Disease	7280 (0.6)					
	Metabolic	3622 (0.3)					
	Vascular	27830 (2.4)					
	Other	764691 (66.2)					
Time to Death/Follow-Up (months) – mean (SD)		3.5 (3.2)	5.4 (4.2)	3.5 (3.2)			

* Abbreviations used: TTP = Thrombotic thrombocytopenic purpura, OR = Odds ratio, CI = Confidence interval, SD = Standard deviation, HD = Hemodialysis, PD = Peritoneal dialysis, NR = Not reported, due to privacy concerns USRDS guidelines do not allow reporting of values ≤ 10, AVF = Arteriovenous fistula

† Note: Black filled cells represent variables that did not remain in the final logistic regression model

Supplementary Table S4. Full and final logistic regression models on TTP plus treatment†

Variable	Level	Full			Final		
		OR	95% CI	p-value	OR	95% CI	p-value
Age	1-yr increase	0.95	0.94-0.96	<0.0001	0.95	0.94-0.96	<0.0001
Sex	Female vs. Male	1.57	1.23-2.00	0.0013	1.59	1.25-2.02	0.0002
Race	Black vs. White	0.67	0.51-0.89	0.0193	0.67	0.51-0.89	0.0194
	Other vs. White	0.95	0.59-1.54		0.95	0.59-1.54	
Ethnicity	Hispanic vs. Non-Hispanic	0.43	0.28-0.66	0.0001	0.43	0.28-0.66	0.0001
Access Type	Catheter vs. AVF	1.12	0.78-1.59	0.8270			
	Graft vs. AVF	1.04	0.48-2.25				
Illicit Drug Use	Yes vs. No	1.02	0.53-1.96	0.9563			
Tobacco Use	Yes vs. No	2.09	1.58-2.77	<0.0001	2.08	1.58-2.75	<0.0001
Alcohol Dependent	Yes vs. No	0.87	0.49-1.54	0.6316			
CCI	1 unit change	1.13	1.10-1.17	<0.0001	1.13	1.10-1.17	<0.0001

†Note due to low frequencies, dialysis modality and hypothyroidism could not be examined in full or final logistic regression models. Cells that are shaded black were eliminated from the final multivariable logistic regression model. Abbreviations used: TTP=thrombotic thrombocytopenic purpura, OR=odds ratio, CI=confidence interval, AVF=arteriovenous fistula, CCI= Charlson Comorbidity Index

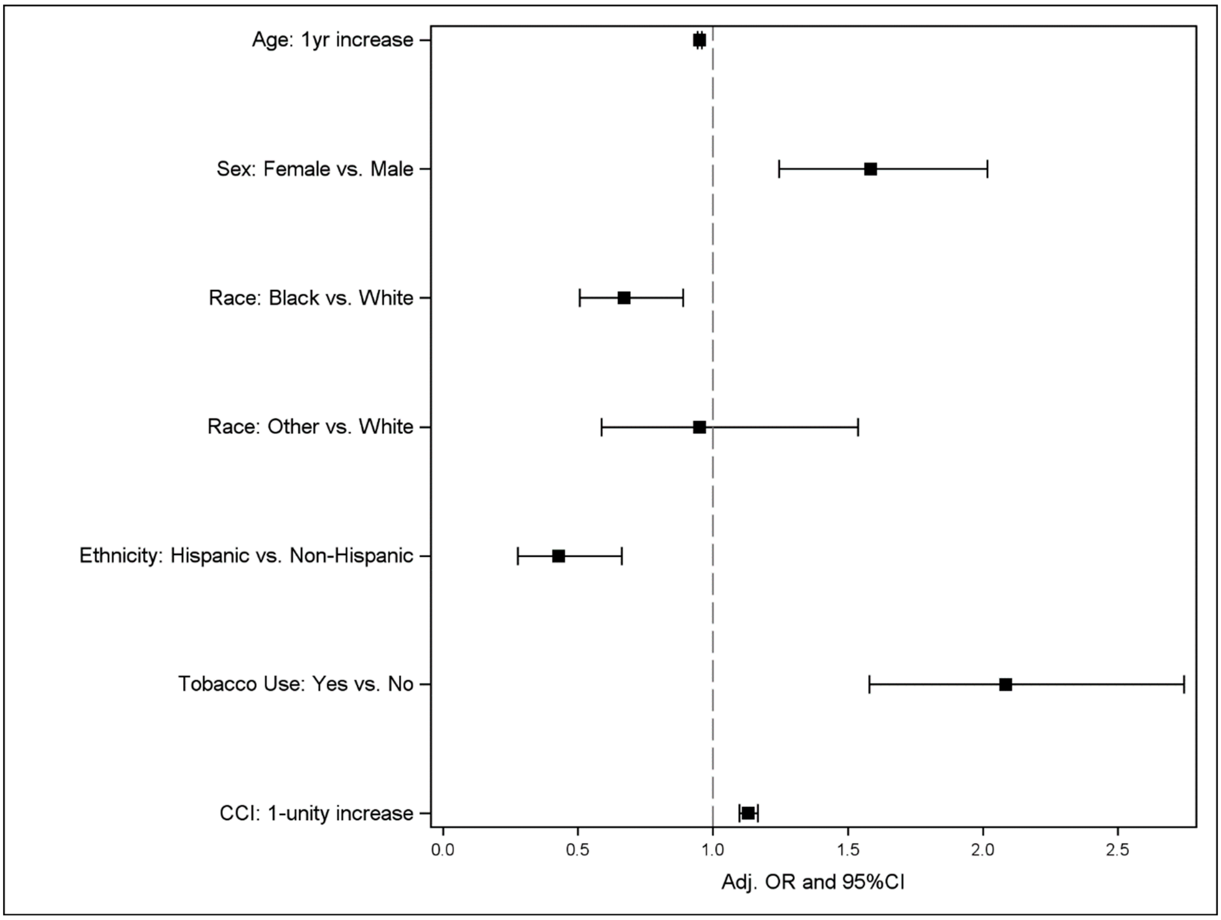

Supplementary Figure S1. Forest plot of final multiple logistic regression model on TTP plus treatment. Abbreviation used: CCI=Charlson Comorbidity Index
